# Epitopes recognised by neutralising and non-neutralising anti-envelope antibodies define surface exposed regions of HTLV-1 envelope

**DOI:** 10.1186/1742-4690-11-S1-P81

**Published:** 2014-01-07

**Authors:** David W Brighty, Mariluz Arainga, Chien-Wen S Kuo, Abigail Fernades

**Affiliations:** 1 Division of Cancer Research, Medical Research Institute, University of Dundee, Scotland, UK

## 

Infection of human cells by human T cell lymphotropic virus type 1 (HTLV-1) is mediated by the viral envelope glycoproteins. The gp46 surface glycoprotein binds to cell surface receptors, including heparan sulfate proteoglycans, neuropilin 1, and glucose transporter 1, allowing the transmembrane glycoprotein (TM, gp21) to initiate fusion of the viral and cellular membranes. The envelope glycoproteins are recognized by neutralizing Abs and CTL following a protective immune response and, therefore, represent attractive components for a HTLV-1 vaccine. Recently, we demonstrated that recombinant forms of surface glycoprotein (sRgp46) or TM are capable of eliciting polyclonal antibody responses following vaccination in a murine model. Analysis of monoclonal antibodies derived from these animals indicates that they exhibit properties that are typical of those observed in natural human infections. We now demonstrate that neutralizing antibodies appear to recognize a limited set of surface accessible neutralising epitopes. We have mapped some of these epitopes at amino acid resolution and demonstrate that several overlapping epitopes within the receptor-binding domain of SU define a hotspot for recognition by neutralising antibodies. Surprisingly, some non-neutralising monoclonal antibodies also appear to recognise surface exposed epitopes of SU; models for sensitivity to neutralisation will be discussed. Finally, we demonstrate that many HTLV-1 infected patients direct antibodies to gp21 TM, but this activity is largely unable to block viral infectivity. The implications of our findings for HTLV-1 pathogenesis and vaccine development will be discussed.

